# Magnesium Alleviates Adverse Effects of Lead on Growth, Photosynthesis, and Ultrastructural Alterations of *Torreya grandis* Seedlings

**DOI:** 10.3389/fpls.2016.01819

**Published:** 2016-11-30

**Authors:** Jie Shen, Lili Song, Karin Müller, Yuanyuan Hu, Yang Song, Weiwu Yu, Hailong Wang, Jiasheng Wu

**Affiliations:** ^1^The Nurturing Station for the State Key Laboratory of Subtropical Silviculture, Zhejiang A & F UniversityZhejiang, China; ^2^New Zealand Institute for Plant and Food Research Limited, Ruakura Research CentreHamilton, New Zealand; ^3^Key Laboratory of Soil Contamination Bioremediation of Zhejiang Province, Zhejiang A & F UniversityZhejiang, China

**Keywords:** *Torreya grandis*, lead toxicity, magnesium, heavy metal phytotoxicity, phytoremediation

## Abstract

Magnesium (Mg^2+^) has been shown to reduce the physiological and biochemical stress in plants caused by heavy metals. To date our understanding of how Mg^2+^ ameliorates the adverse effects of heavy metals in plants is scarce. The potential effect of Mg^2+^ on lead (Pb^2+^) toxicity in plants has not yet been studied. This study was designed to clarify the mechanism of Mg^2+^-induced alleviation of lead (Pb^2+^) toxicity. *Torreya grandis* (*T. grandis*) seedlings were grown in substrate contaminated with 0, 700 and 1400 mg Pb^2+^ per kg^-1^ and with or without the addition of 1040 mg kg^-1^ Mg^2+^. Growth parameters, concentrations of Pb^2+^ and Mg^2+^ in the plants’ shoots and roots, photosynthetic pigment, gas exchange parameters, the maximum quantum efficiency (Fv/Fm), root oxidative activity, ultrastructure of chloroplasts and root growth were determined to analyze the effect of different Pb^2+^ concentrations on the seedlings as well as the potential ameliorating effect of Mg^2+^ on the Pb^2+^ induced toxicity. All measurements were tested by a one-way ANOVA for the effects of treatments. The growth of *T. grandis* seedlings cultivated in soils treated with 1400 mg kg^-1^ Pb^2+^ was significantly reduced compared with that of plants cultivated in soils treated with 0 or 700 mg kg^-1^ Pb^2+^. The addition of 1040 mg kg^-1^ Mg^2+^ improved the growth of the Pb^2+^-stressed seedlings, which was accompanied by increased chlorophyll content, the net photosynthetic rate and Fv/Fm, and enhanced chloroplasts development. In addition, the application of Mg^2+^ induced plants to accumulate five times higher concentrations of Pb^2+^ in the roots and to absorb and translocate four times higher concentrations of Mg^2+^ to the shoots than those without Mg^2+^ application. Furthermore, Mg^2+^ addition increased root growth and oxidative activity, and protected the root ultrastructure. To the best of our knowledge, our study is the first report on the mechanism of Mg^2+^-induced alleviation of Pb^2+^ toxicity. The generated results may have important implications for understanding the physiological interactions between heavy metals and plants, and for successful management of *T. grandis* plantations grown on soils contaminated with Pb^2+^.

## Introduction

Heavy metal pollution has become a global environmental threat (Krabbenhoft and Sunderland, 2013; Chen et al., 2015). Among metal contaminants, lead (Pb^2+^) is a major concern because of its extensive distribution in the environment and the substantial environmental and human health problems it can cause. Major sources of Pb^2+^ pollution include mining and smelting activities as well as Pb^2+^-containing paints, gasoline, explosives, sewage sludge and fertilizers (Sharma and Dubey, 2005; Buekers et al., 2009). When plants are exposed to Pb^2+^, even at micromolar levels, adverse effects can occur on plant growth (Hadi et al., 2010), root elongation (Liu et al., 2000), seed germination (Lamhamdi et al., 2011), seedling development (Kaur et al., 2015), chlorophyll production (Rashid and Popovic, 1990), chloroplast lamellar organization (Hu et al., 2007), and antioxidant enzymes system (Gupta et al., 2009, 2010). However, the toxicological response to Pb^2+^ varies depending on the plant species and tissues analyzed (Pourrut et al., 2011). Huang and Cunningham (1996) showed significant differences in the uptake and translocation of Pb^2+^ among *Triticum aestivum, Thlaspi rotundifolium*, and *Thlaspi caerulescens. Mimosa caesalpiniaefolia* was more tolerant to high Pb^2+^ concentrations in soil than *Erythrina speciosa* and *Schizolobium parahyba* (de Souza et al., 2012). Huang and Cunningham (1996) found that some dicot species can accumulate significantly higher concentrations of Pb^2+^ in the roots than some monocot species.

Heavy metals could be taken up by cation transporters such as members of the ZIP (Zn-regulated transporter/Fe-regulated transporter-like protein) and natural resistance-associated macrophage protein families (Eide et al., 1996; Korshunova et al., 1999; Guerinot, 2000; Thomine et al., 2000). These bivalent cation transporters are also important uptake systems for essential elements. Therefore, nutrients such as magnesium (Mg^2+^) are considered to contribute to plants’ tolerance to heavy metal exposure owing to their chemical similarity as well as sharing common transporters with heavy metals (Guerinot, 2000; Pittman, 2005). Over the last decade, studies have revealed the ability of Mg^2+^ to mitigate heavy metal toxicity caused by aluminum (Al^3+^) and cadmium (Cd^2+^) (Kashem and Kawai, 2007; Bose et al., 2011). Kashem and Kawai (2007) reported that adding Mg^2+^ to nutrient solutions reduced Cd concentrations in plants and enhanced the growth of plants suffering from Cd toxicity. Hermans et al. (2011) indicated that the protective effect of Mg^2+^ against Cd toxicity may be at least partly attributed to the protection of the photosynthetic apparatus. However, only few studies have investigated the effect of Mg^2+^ on Pb^2+^ toxicity. Therefore, we explored the effect of Mg^2+^ on Pb^2+^ toxicity and the possible mechanism of Mg^2+^-induced alleviation of Pb^2+^ toxicity using a local species *Torreya grandis* (*T. grandis*).

*Torreya grandis* is a gymnosperm tree species belonging to the Taxaceae family, mainly grown in eastern China with significant economic value because of its valuable drupe-like fruits with medicinal effects from its anthelmintic, antitussive, carminative, laxative, antifungal, antibacterial, and antitumor properties (Huang et al., 2001). As the demand for the fruit increased, the acreage of *T. grandis* has rapidly expanded, and the management intensity has increased with higher inputs of fertilizers and pesticides, such as lead arsenate. In addition, soils near highways, which are usually polluted by exhaust emissions, have also been used for growing *T. grandis*. Therefore, *T. grandis* is likely to face with Pb^2+^ stress. It remains unclear whether *T. grandis* can be tolerant to high level of Pb^2+^ stress. Thus, in this study, we performed a pot experiment to test the following hypotheses: (1) High level Pb^2+^ stress inhibits the growth of *T. grandis* seedlings; (2) Mg^2+^ can effectively ameliorate the negative effects of lead stress on the growth of *T. grandis* seedlings. The information obtained in this study is valuable for the propagation and cultivation of *T. grandis* under Pb^2+^ stress conditions.

## Materials and Methods

### Plant Material and Growth Conditions During All Experiments

All experiments were conducted on the Zhejiang A & F University campus, Lin’an City, Zhejiang province (330°23′N, 119°72′E), China. Two-year-old uniform and healthy *T. grandis* seedlings (mean ground diameter 5 ± 0.5 mm and seedling height 35 ± 2 cm) were transplanted into plastic pots (16.5 cm inner diameter, 18 cm height, with holes in the bottom, one seedling per pot) filled with 2.5 kg of sterilized substrate mixture of perlite and quartz sand (1:1, v/v). All pots were irrigated with 200 ml of Hoagland’s nutrient solution (3.0 mM KNO_3_, 2.5 mM Ca(NO_3_)_2_, 1.0 mM MgSO_4_^.^ 7H_2_O, 1.2 μM FeEDTA, 4.0 μM MnCl_2_, 22.0 μM H_3_BO_3_, 0.4 μM ZnSO_4_, 0.05 μM Na_2_MoO_4_, 1.6 μM CuSO_4_, and 1.0 μM KH_2_PO_4_) every three days and maintained at 75% field capacity of the growth substrate to keep the plants well watered. Day and night temperature was kept between 18.0 and 32.0°C and the relative humidity ranged between 50 and 80%. The light intensity in the greenhouse was monitored daily with an external quantum sensor attached to LI-6400 (Li-COR, Lincoln, NE, USA) and kept within the range of 500–800 μmol m^-2^ s^-1^ photosynthetically active radiation (PAR) above the plants.

### First Experiment: Exposure of Seedlings to Pb^2+^

The first experiment to determine the lead concentration that adversely affected *T. grandis* seedlings was carried out between 1 May and 31 June 2014. One month after transplantation of the seedlings, the height and ground diameter of each seedling were measured as reference values. A completely randomized design with three replications per treatment and five plants per replication was chosen. Total of 45 seedlings were exposed to Pb^2+^ supplied as Pb (NO_3_)_2_ at concentrations of 0 (control), 700 and 1400 mg Pb^2+^ kg^-1^ growth substrate. These concentrations were selected based on a report by Huang et al. (2006), who found that Pb^2+^-concentrations in soil exceeding 1000 mg kg^-1^ affected the growth of *Pinus rigida*. After 60 days, the height and ground diameters of the seedlings were recorded.

### Second Experiment: Exposure of Seedlings to Pb^2+^ and Mg^2+^

The above experiment showed that 1400 mg kg^-1^ Pb^2+^ caused Pb^2+^ toxicity in *T. grandis* seedlings. Hence, further studies on the effect of Mg^2+^ on Pb^2+^ toxicity were restricted to the control and the 1400 mg kg^-1^ Pb^2+^ treatments. This second experiment was conducted during 1 May to 31 June 2015. Magnesium (with the irrigation water) was supplied as MgCl_2_ at 1040 mg kg^-1^. In total, the following four treatments (total of 60 seedlings) were established: T1 (control without Mg^2+^ addition); T2 (control with 1040 mg kg^-1^ Mg^2+^); T3 (1400 mg kg^-1^ Pb^2+^ without 1040 mg kg^-1^ Mg^2+^) and T4 (1400 mg kg^-1^ Pb^2+^ with 1040 mg kg^-1^ Mg^2+^). A completely randomized design with three replications per treatment and five plants per replication was set up.

### Plant Harvest

After 60 days of the second experiment, the third and fourth leaves from the plant top, which had been completely developed when Pb^2+^ treatment started, were collected from all plants, cleaned with tissue paper to remove any surface contamination, immediately frozen in liquid nitrogen and stored at -70°C. Plant growth, concentrations of Pb^2+^ and Mg^2+^ in shoots and roots, chlorophyll concentration, root oxidative activity, photosynthesis and ultrastructure of chloroplasts and roots were determined for all samples.

### Growth and Morphology Analysis

After 60 days of the two experiments, all seedlings were harvested and separated into shoots and roots for growth and morphology analyses. Shoot biomass and total biomass were measured after drying of the shoots and roots at 80°C for 4 days. Seedling height was defined as the height of the plant from the top of the growth medium to the tip of the uppermost shoot.

### Determination of Pb^2+^ and Mg^2+^ Concentrations in Plant Shoots and Roots

To determine the concentrations of Pb^2+^ and Mg^2+^ in the shoots and roots, the dried plant materials were grounded with a stainless steel mill and passed through a 0.25 mm sieve for analysis of Pb^2+^ and Mg^2+^. An aliquot of 0.1 g of the dried plant materials of each treatment was digested with HNO_3_–HClO_4_ (4:1, v/v), and the digest was diluted with deionized water (DW) to a final volume of 50 mL. Concentrations of Pb^2+^ and Mg^2+^ in the filtrates were analyzed by flame atomic absorption spectroscopy (Perkin Elmer Analyser 300, England). The Pb^2+^ and Mg^2+^ concentrations in the entire plant were calculated following Zhang et al. (2011) and expressed in mg kg^-1^ DW and mg g^-1^ DW, respectively.

### Pigment Concentration in Leaves

Approximately 0.1 g of finely cut and well-mixed fresh plant sample, which was taken from healthy and fully developed leaves at the same position in each treatment, was repeatedly extracted with 8 mL of 95% ethanol (100%, Sinopharm Chemical Reagent Company, Shanghai, China). Pigment was extracted at 4°C for 24 h in darkness and shaken three or four times until the leaf samples blanched (no green color in the leaf tissue). The absorbance was measured with a Shimadzu UV-2550 spectrophotometer (Kyoto, Japan) at 664, 649, and 470 nm after centrifugation of the mixture. The chlorophyll a (Chla), chlorophyll b (Chlb), total chlorophyll (Chl(a+b)), and carotenoid (Car) contents were calculated using the following formulas (Lichtenthaler, 1987). Results are expressed in mg g^-1^ fresh weight (FW).

(1) $$\begin{array}{c} C_{a}\left(gL^{-1}\right)=13.36A_{664}-5.19A_{649} \end{array}$$

(2) $$\begin{array}{c} C_{b}\left(gL^{-1}\right)=27.43A_{649}-5.10A_{664} \end{array}$$

(3) $$\begin{array}{c} C_{a+b}\left(gL^{-1}\right)=5.24A_{664}-22.24A_{649} \end{array}$$

(4) $$\begin{array}{c} Cx+c\left(gL^{-1}\right)=\frac{1000A_{470}-2.13C_{a}-97.64C_{b}}{209} \end{array}$$

Where, C*_a_*, C*_b_*, C*_a+b_*, and C*_x+c_* were the concentrations of Chla, Chlb, Chl (a+b), and Car, respectively. *A*_664_, *A*_649_, and *A*_470_ were the absorbances of pigment extract solution at 664, 649, and 470 nm wavelengths, respectively.

### Photosynthetic Parameters and the Maximum Quantum Efficiency of Psii Photochemistry (Fv/Fm)

The youngest healthy and fully developed leaves randomly selected from the first branch were chosen for gas exchange measurements. Field gas exchange measurements were conducted with a LI-6400 portable photosynthesis system (Li-COR, Inc. Lincoln, NE, USA) with a standard leaf chamber equipped with a 6400-02B LED light source (LI-6400, Li-COR, Lincoln, NE, USA). Measurements were conducted at an air concentration of 21% O_2_, 400 μmol mol^-1^ carbon dioxide (CO_2_), 800 μmol m^-2^ s^-1^ PAR, 50% relative humidity and a temperature of 20 ± 2°C. The gas exchange measurements were performed on sunny days from 8:30 to 11:30 am.

Chlorophyll fluorescence (Fv/Fm) was determined in the morning (08:00 am–11:00 am) on the healthy and fully developed leaves with a pulse modulation fluorometer (PAM-2500, Walz, Effeltrich, Germany). After 30 min of adaptation to the dark (Tang et al., 2015), the minimum fluorescence (Fo) was determined in a measuring light of approximately 0.5 μmol photon m^-2^ s^-1^, and the maximum fluorescence (Fm) was determined under a 0.8-s saturating flash of 10,000 μmol photon m^-2^ s^-1^. The Fv/Fm value was calculated as (Fm-Fo)/Fm (Maxwell and Johnson, 2000).

### Determination of Root Morphological Traits

After gently washing the roots with deionized water, the total length, volume, and surface area of the root samples were determined by image analysis. The roots were photographed and then the images were analyzed with the root image analysis system software WinRHIZO ^1^.

### Root Oxidative Activity

The root oxidative activity was measured according to the method of Mishra (2012) with a slight modification. About 3 g fresh root were immersed in 300 ml of 20 ppm á-naphthylamine solution for 10 minutes to exclude any initial rapid absorption of á-naphthylamine by roots. The intact roots were then transferred to another vial with 300 ml of 20 ppm of á-naphthylamine solution and incubated for four hours at 25 ± 1°C. Then, 2 ml of the incubated solution were mixed with 10 ml of 0.1% sulfanilic acid (in 3% acetic acid) and 2 ml of 50 ppm NaNO_2_, and diluted to 25 ml using distilled water. The absorbance of the colored solution was determined at 530 nm using spectrophotometry. Root oxidative activity was expressed as μg á-naphthylamine h^-1^ g^-1^ FW.

### Ultrastructure of Chloroplast and Root

To examine the chloroplast ultrastructure of mesophyll cells, fresh leaves were immediately fixed in 2.5% (v/v) glutaraldehyde (0.1 mol L^-1^ phosphate buffer, pH 7.2) for at least 48 h after detachment from the plants. The samples were immersed in 1% (v/v) osmium acid for post-fixation, embedded in resin, and ultrathin sectioned for transmission electron microscopy (H7650, Hitachi, Tokyo, Japan).

### Data Analysis

Because the Pb^2+^ and Mg^2+^ treatments were not applied independently to each seedling, the plants in each treatment combination were not true replicates (Hurlbert, 1984; Maherali and DeLucia, 2000). Therefore, averages of subsamples (five seedlings per replicate) were used for the analysis of variance. All measurements were tested by a one-way ANOVA for the effects of treatments (combinations of Pb^2+^ and Mg^2+^ concentration). The effects were considered significant at *P* < 0.05. Before ANOVA, data were checked for normality and homogeneity of variances, and log-transformed to correct deviations from these assumptions when needed. Significant differences among treatment means were analyzed using Tukey’s multiple comparison post hoc tests. The used statistical software package was SPSS 16 for Windows (SPSS Inc., Chicago, IL, USA).

## Results

### Effect of Lead on Plant Growth and Development

**FIGURE 1:**
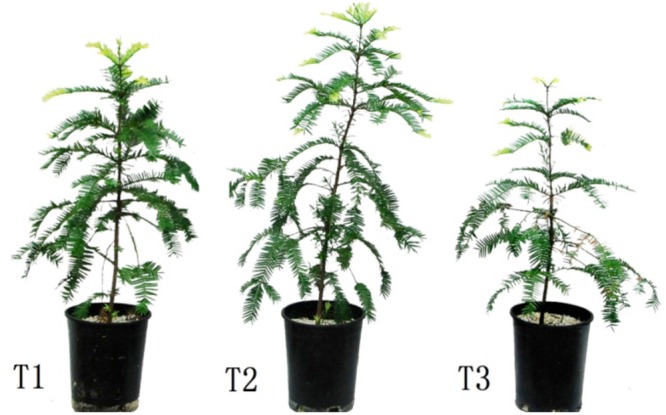
**Effect of Pb^2+^ addition to the growth medium on *T. grandis* seedlings.** T1, control, T2, 700 mg kg^-1^ Pb^2+^, T3, 1400 mg kg^-1^ Pb^2+^.

**FIGURE 2:**
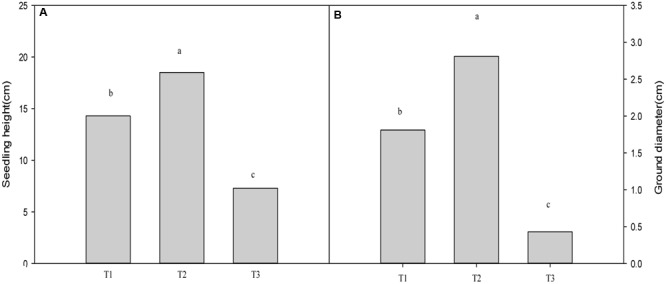
**Effect of Pb^2+^ addition to growth medium on the seedling height (A)** and ground diameter **(B)** of *T. grandis* seedlings. T1, control, T2, 700 mg kg^-1^ Pb^2+^, T3, 1400 mg kg^-1^ Pb^2+^. Data points and error bars represent *mean* ± *standard deviation* (*n* = 3). Different lower case letters above the columns indicate significant (*P* < 0.05) difference between treatments.

Plants grown for 60 days at 0, 700, and 1400 mg Pb^2+^ per kg^-1^ soil could be visually differentiated. Plants grown at 700 mg kg^-1^ were larger than those of other treatments (**Figure 1**). Compared with the control, soil contamination of 700 mg Pb^2+^ kg^-1^ significantly increased the growth of *T. grandis* seedlings (*P* = 0.0001, **Figure 2**). However, the 1400 mg kg^-1^ Pb^2+^ treatment inhibited plant growth and ground diameter by 60.5% (*P* = 0.0001) and 83.0% (*P* = 0.0001), respectively (**Figure 2**).

### Effect of Mg^2+^ on Dry Biomass, Plant Growth, and Morphological Traits of Roots Under Lead Toxicity

Compared with the control seedlings, exposure of plants to 1400 mg kg^-1^ Pb^2+^ in a growth medium for 60 days significantly decreased the dry mass of shoots and roots by 19.6% (*P* = 0.0002) and 24.1% (*P* = 0.0038), respectively (**Table 1**). The Mg^2+^-treated plants had significantly higher shoot (*P* = 0.0172) and root (*P* = 0.0118) dry mass than plants under Pb^2+^ toxicity without Mg^2+^ application (**Table 1**). However, Mg^2+^ had no significant effect on the dry mass of shoots (*P* = 0.5937) and roots (*P* = 0.9235) of the non-Pb^2+^-stressed plants. The leaf area (*P* = 0.0001) and seedling height (*P* = 0.0001) of plants under Pb^2+^ toxicity were significantly lower than those of the control plants. Application of Mg^2+^ increased the leaf area (*P* = 0.0004) and seedling height (*P* = 0.0001) of plants under Pb^2+^ toxicity (*P* < 0.05, **Table 1**). However, there were no significant differences in leaf area (*P* = 0.4141) and seedling height (*P* = 0.1411) in the non-lead-stressed plants treated with or without Mg^2+^.

**Table 1:** Effects of Mg^2+^ on the dry biomass of shoots and roots, leaf area (LA), seedling height, and root morphological traits of *T. grandis* seedlings grown under Pb^2+^ toxicity.

Treatment	Shoot Biomass (g)	Root Biomass (g)	LA (cm^2^)	Seedling height (cm)	Total root length (cm)	Root surface area (cm^2^)	Root volume (cm^3^)
T1	26.0 ± 1.20^a^	11.6 ± 0.64^a^	0.7 ± 0.04^ab^	14.3 ± 0.17^a^	1614.6 ± 70.54^a^	865.2 ± 7.42^a^	38.8 ± 0.41^a^
T2	26.7 ± 0.35^a^	11.9 ± 0.21^a^	0.8 ± 0.02^a^	14.8 ± 0.15^a^	1682.1 ± 7.78^a^	919.7 ± 5.45^a^	41.9 ± 0.39^a^
T3	20.9 ± 0.35^c^	8.8 ± 0.49^b^	0.5 ± 0.02^c^	7.3 ± 0.10^c^	1179.7 ± 128.0^b^	616.4 ± 8.48^c^	25.8 ± 2.34^c^
T4	23.4 ± 0.78^b^	11.1 ± 1.48^a^	0.7 ± 0.01^b^	10.8 ± 0.15^b^	1506.8 ± 23.74^a^	766.8 ± 14.90^b^	32.0 ± 2.50^b^

*Data points and error bars represent *mean* ±*standard deviation* of three replicates. Different letters indicate significant differences (*P* < 0.05); *n* = 3. The same letter in the same column denotes no significant difference among treatments. Treatments: T1, control; T2, control + 1040 mg kg^-1^ Mg^2+^; T3, 1400 mg kg^-1^ Pb^2+^; T4, 1400 mg kg^-1^ Pb^2+^ + 1040 mg kg^-1^ Mg^2+^.*

Total length, surface area and volume of plant roots under Pb^2+^ toxicity decreased significantly by 26.9% (*P* = 0.0004), 28.8% (*P* = 0.0001), and 33.5% (*P* = 0.0001), respectively, compared with the control plants (**Table 1**). Treatment of Pb^2+^-stressed plants with Mg^2+^ significantly increased total length, surface area and volume of roots by 27.7% (*P* = 0.0029), 24.3% (*P* = 0.0113), and 24.0% (*P* = 0.0001), respectively, compared with plants treated only with Pb^2+^ for 60 days.

### Effect of Mg^2+^ on Photosynthetic Pigments and Gas Exchange Parameters of Plants Under Lead Toxicity

Variations in the levels of photosynthetic pigments, including chlorophyll a (Chla), chlorophyll b (Chlb), and carotenoids (Car), were evaluated in *T. grandis* seedlings under lead toxicity (**Table 2**). The Chla (*P* = 0.0001) concentrations, Chlb (*P* = 0.0001) concentrations, Car (*P* = 0.0004) concentrations and Chla/Chlb (*P* = 0.0003) ratios were lower but the Car/Chl(a+b) (*P* = 0.0003) ratios were higher in the Pb^2+^-treated plants than in the control plants. Application of Mg^2+^ resulted in higher Chla (*P* = 0.0001), Chlb (*P* = 0.0001), and Car (*P* = 0.0019) concentrations, and also increased the Chla/b (*P* = 0.0012) ratios but lowered the Car/Chl(a+b) (*P* = 0.0005) ratios in the leaves of plants exposed to 1400 mg kg^-1^ Pb^2+^ (**Table 2**). However, significant difference in chlorophyll and carotenoid concentrations between seedlings treated with and without Mg^2+^ under no Pb^2+^ toxicity was not found.

**Table 2:** Effects of Mg^2+^ on chlorophyll a (Chla), chlorophyll b (Chlb), carotenoids (Car), Chl(a+b), chlorophyll a:b ratio (Chla/Chlb), and Car/ Chl(a+b) of *T. grandis* seedling leaves under Pb^2+^ toxicity.

Treatment	Chla (mg/g)	Chlb (mg/g)	Car (mg/g)	Chl (a+b) (mg/g)	Chla/Chlb	Car/Chl(a+b)
T1	1.0 ± 0.01^a^	0.5 ± 0.01^a^	0.3 ± 0.01^a^	1.5 ± 0.01^a^	2.1 ± 0.02^a^	0.2 ± 0.01^b^
T2	1.0 ± 0.03^a^	0.5 ± 0.02^a^	0.3 ± 0.01^a^	1.5 ± 0.04^a^	2.1 ± 0.07^a^	0.2 ± 0.02^b^
T3	0.5 ± 0.02^c^	0.3 ± 0.01^b^	0.2 ± 0.02^b^	0.8 ± 0.02^c^	1.8 ± 0.02^c^	0.3 ± 0.03^a^
T4	0.9 ± 0.04^b^	0.5 ± 0.02^a^	0.3 ± 0.02^a^	1.4 ± 0.03^b^	2.0 ± 0.04^b^	0.2 ± 0.01^b^

*Data points and error bars represent *mean* ±*standard deviation* of three replicates. Different letters indicate significant differences (*P* < 0.05); *n* = 3. The same letter in the same column denotes no significant difference among treatments. Treatments: T1, control; T2, control + 1040 mg kg^-1^ Mg^2+^; T3, 1400 mg kg^-1^ Pb^2+^; T4, 1400 mg kg^-1^ Pb^2+^ + 1040 mg kg^-1^ Mg^2+^.*

**FIGURE 3:**
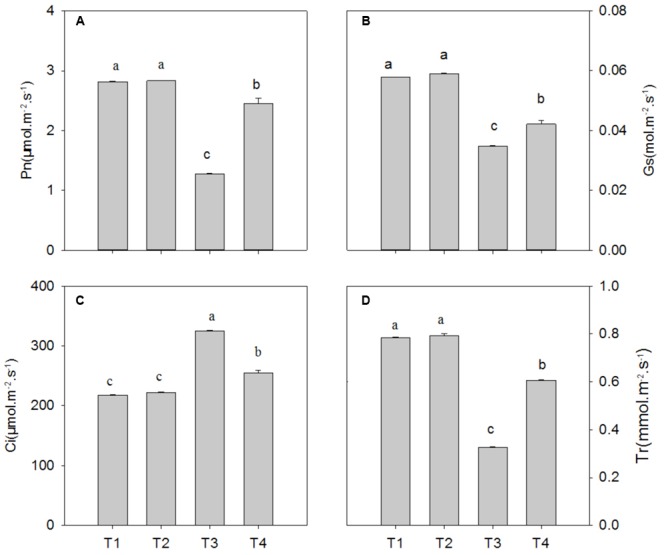
**The net photosynthetic rate (Pn) (A)**, stomatal conductance (Gs) **(B)**, internal carbon dioxide concentration (Ci) **(C)**, and transpiration rate (Tr) **(D)** of *T. grandis* seedlings grown in media amended with various amounts of Pb^2+^ and Mg^2+^. Treatments: T1, control; T2, control + 1040 mg kg^-1^ Mg^2+^; T3, 1400 mg kg^-1^ Pb^2+^; T4, 1400 mg kg^-1^ Pb^2+^ + 1040 mg kg^-1^ Mg^2+^. Error bars are standard deviation (*n* = 3). Different lower case letters above the columns indicate significant (*P* < 0.05) difference between treatments.

Compared with the control, Pb^2+^ toxicity significantly decreased the photosynthetic rate (Pn), stomatal conductance (Gs) and transpiration (Tr) by 54.6% (*P* = 0.0001), 39.8% (*P* = 0.0001), and 58.4% (*P* = 0.0001), respectively, while it increased intercellular CO_2_ (Ci) by 49.1% (*P* = 0.0001) (**Figure 3**; *P* < 0.05). In leaves of plants under Pb^2+^ toxicity, Mg^2+^ treatment significantly increased the levels of Pn, Gs, and Tr by 91.8% (*P* = 0.0001), 21.0% (*P* = 0.0001), and 86.4% (*P* = 0.0001), respectively, whereas it decreased Ci levels by 21.4% (*P* = 0.0001) compared with non-Mg^2+^-treated plants under Pb^2+^ toxicity (**Figure 3**).

### Effect of Mg^2+^ on Chlorophyll Fluorescence and Oxidative Activity of Roots in Plants Under Lead Toxicity

**FIGURE 4:**
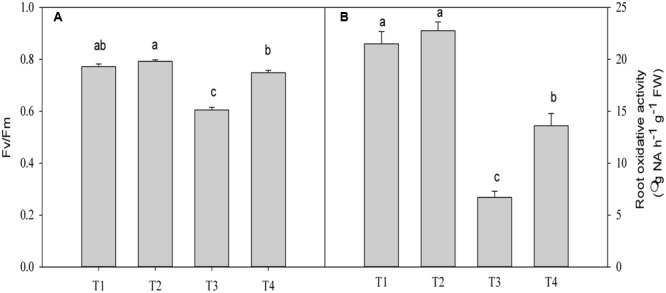
**The maximum quantum efficiency (Fv/Fm) (A)** and root oxidative activity **(B)** of *T. grandis* seedlings grown in media amended with various amounts of Pb^2+^ and Mg^2+^. Treatments: T1, control; T2, control + 1040 mg kg^-1^ Mg^2+^; T3, 1400 mg kg^-1^ Pb^2+^; T4, 1400 mg kg^-1^ Pb^2+^ + 1040 mg kg^-1^ Mg^2+^. Error bars are standard deviation (*n* = 3). Different lower case letters above the columns indicate significant (*P* < 0.05) difference between treatments.

The Fv/Fm value was significantly decreased by 21.6% (*P* = 0.0001) in plants under Pb^2+^ toxicity compared with the control (**Figure 4B**). Application of Mg^2+^ significantly increased the Fv/Fm value by 23.7% (*P* = 0.0001)in leaves of seedlings exposed to 1400 mg kg^-1^ Pb^2+^ (**Figure 4A**).

The root oxidative activity in plants under Pb^2+^ toxicity decreased compared with the control (*P* = 0.0001, **Figure 4B**). Application of Mg^2+^ to Pb^2+^-stressed plants significantly increased root oxidative activity by 102.9% (*P* = 0.0001) compared with plants only treated with Pb^2+^ (**Figure 4A**). However, significant difference in oxidative activity between plants treated with and without Mg under no Pb^2+^ toxicity was not found (*P* = 0.4361, **Figure 4B**).

### Effect of Mg^2+^ on Pb^2+^ and Mg^2+^ Accumulation in Plant Tissues Under Lead Toxicity

Under Pb^2+^ toxicity, Pb^2+^ contents in the roots were three times higher than in the shoots, indicating that the roots accumulated the majority of the absorbed Pb^2+^. After application of Mg^2+^, the Pb^2+^ concentrations in the roots were higher than in roots of the Pb^2+^-stressed plants, whereas the Pb^2+^ uptake in the above-ground parts was lower compared to the plants without Mg^2+^ application. Interestingly, the distribution of Mg^2+^ in roots and shoots of the *T. grandis* seedlings differed significantly. The Mg^2+^ concentration in the shoots of the control plants was higher than in the roots. However, Pb^2+^ toxicity had no significant effect on the distribution of Mg^2+^ between roots (*P* = 0.6412) and shoots (*P* = 0.8785) compared with the control. Application of Mg^2+^ significantly increased Mg^2+^ accumulation in the shoots (*P* = 0.0001) and roots (*P* = 0.0002), and Mg^2+^ concentration was four times higher in the shoots than in the roots (**Table 3**).

**Table 3:** Effects of Mg^2+^ on concentrations of Pb^2+^ and Mg^2+^ in shoots and roots of *T. grandis* seedlings grown under Pb^2+^ toxicity.

Treatment	Pb^2+^ content (mg kg^-1^) Root	Shoot	Mg^2+^ content (mg g^-1^) Root	Shoot
T1	n.d.	n.d.	0.39 ± 0.03^b^	0.74 ± 0.03^b^
T2	n.d.	n.d.	0.73 ± 0.08^a^	3.27 ± 0.05^a^
T3	689.1 ± 30.5^b^	231.4 ± 12.3^a^	0.44 ± 0.05^b^	0.79 ± 0.02^b^
T4	876.1 ± 20.2^a^	156.2 ± 3.6^b^	0.76 ± 0.01^a^	3.37 ± 0.2^a^

*Data points and error bars represent mean ± standard deviation of three replicates. Different letters indicate significant differences (*P* < 0.05); *n* = 3. The same letter in the same column denotes no significant difference among treatments, n.d. = not determined. Treatments: T1, control; T2, control + 1040 mg kg^-1^ Mg^2+^; T3, 1400 mg kg^-1^ Pb^2+^; T4, 1400 mg kg^-1^ Pb^2+^ + 1040 mg kg^-1^ Mg^2+^.*

### Effect of Mg^2+^ on Ultrastructural Modifications of Leaves and Roots in Plants Under Lead Toxicity

**FIGURE 5:**
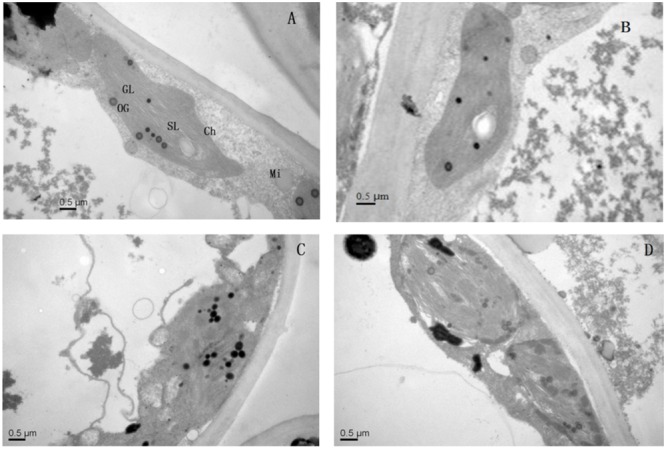
**The transmission electron micrographs of chloroplasts in *T. grandis* seedlings grown in media amended with various amounts of Pb^2+^ and Mg^2+^. (A)** Control; **(B)** control + 1040 mg kg^-1^ Mg^2+^; **(C)** 1400 mg kg^-1^ Pb^2+^; **(D)** 1400 mg kg^-1^ Pb^2+^ + 1040 mg kg^-1^ Mg^2+^.

Application of Mg^2+^ caused significant differences in the ultrastructure of the chloroplasts of the *T. grandis* seedlings grown under Pb^2+^ toxicity (**Figure 5**). Elliptical-shaped chloroplasts with thylakoids were found in the control plants. However, the integrity of the ultrastructure was severely affected by Pb^2+^ toxicity. Chloroplasts were swollen and had irregularly shaped grana, decreased lamellae, and increased osmiophilic granule numbers, and the thylakoid membrane system in plants was in disorder. Interestingly, application of Mg^2+^ promoted the development of chloroplasts, grana and stroma lamellae as well as reduced the osmiophilic granule numbers.

**FIGURE 6:**
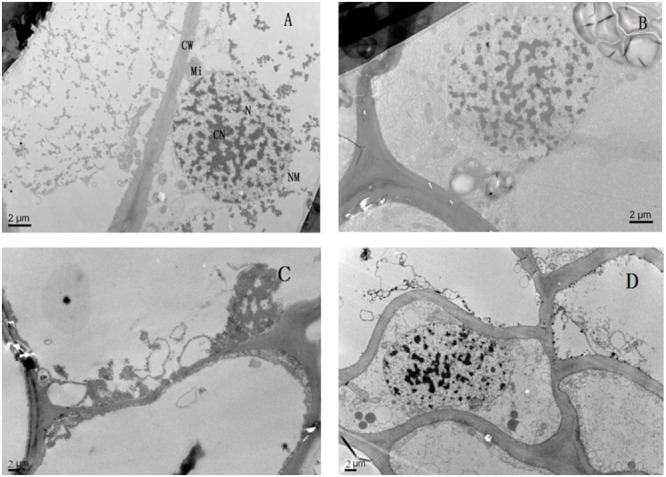
**The transmission electron micrographs of root of *T. grandis* seedlings grown in media amended with various amounts of Pb^2+^ and Mg^2+^. (A)** Control; **(B)** control + 1040 mg kg^-1^ Mg^2+^; **(C)** 1400 mg kg^-1^ Pb^2+^; **(D)** 1400 mg kg^-1^ Pb^2+^ + 1040 mg kg^-1^ Mg^2+^.

Lead toxicity had a marked influence on the ultrastructure of the seedlings’ roots (**Figure 6**). Compared with the control, the root cell structure under lead toxicity was completely destroyed. The nucleus was almost invisible and the mitochondria appeared as hollow bubbles. Application of Mg^2+^ protected the integrity of the root cells as evidenced by visible nuclei, slightly condensed chromatin and irregularly swollen mitochondria with fractured and fuzzy cristae.

## Discussion

The growth of *T. grandis* seedlings was highest at 700 mg kg^-1^ Pb^2+^ in soil, whereas the lowest growth of plants was found at 1400 mg kg^-1^ Pb^2+^ (**Figures 1** and **2**), indicating that lead stress toxicity in *T. grandis* seedlings did not occur at 700 mg kg^-1^ Pb^2+^ in soil. Meanwhile, visible toxic symptoms, such as old leaves yellowing and chlorosis, were observed in plants exposed to 1400 mg kg^-1^ Pb^2+^ (**Figure 1**). The treatment with 1400 mg kg^-1^ Pb^2+^ significantly decreased plant growth and the development of the *T. grandis* seedlings, as indicated by the decreased shoot dry mass, root dry mass, seedling height and leaf area (**Table 1**). Similarly, Hadi et al. (2010) found that 500 mg kg^-1^ Pb^2+^ in soil did not affect the germination rate of maize (*Zea mays*) seeds and that the young maize seedlings did not exhibit any visible toxic symptoms.

Chlorophyll fluorescence is the focus in studies of photosynthetic regulation and plant responses to the environment due to its sensitivity, convenience and non-destructive characteristics (Dai et al., 2009). Generally, plants subjected to heavy metal stress typically have lower Fv/Fm values than non-stressed plants, which is associated with photoinhibition of PSII (Krause and Weis, 1991; Wu et al., 2014). In the present study, the Fv/Fm ratio was significantly reduced in the plants treated with 1400 mg kg^-1^ Pb^2+^ (**Figure 4A**). Indeed, this result was consistent with the gas exchange results, in which lead toxicity decreased Pn, Tr and Gs compared with the control (**Figure 3**), indicating photoinhibition of the photosynthetic capacity in *T. grandis* seedlings under lead stress conditions. A similar result was also described by Rashid and Popovic (1990) for spinach leaves treated with 2 mM Pb^2+^. Interestingly, in this study the Ci value was higher in Pb^2+^-treated plants than in the control plants, indicating that the reduction of photosynthesis under lead stress conditions primarily resulted from non-stomatal limitations. Additionally, photoinhibition and reduction of the photosynthetic capacity under lead stress conditions were manifested by changes of leaf chlorophyll contents. This was explained by its important role in photosynthesis and plant growth. In the present study, we found a strong reduction in the levels of Chla, Chlb, total Chl content and carotenoids in plants treated with 1400 mg kg^-1^ Pb^2+^ (**Table 2**), which was consistent with the results of de Souza et al. (2012), who reported that Pb^2+^ exposure led to a reduction of Chla and Chlb contents in leaves as well as a reduction of Car levels. Application of Mg^2+^ to Pb^2+^-stressed plants improved plant growth, which was accompanied with increased chlorophyll contents, Pn levels and Fv/Fm ratios (**Tables 1** and **2**; **Figures 3** and **4A**). Thus, the positive and beneficial effects of Mg^2+^ on the growth of *T. grandis* seedlings might be associated with improving the photosynthetic capacity and alleviating photoinhibition. A similar result was reported by Hermans et al. (2011), who indicated that the protective effect of Mg^2+^ against Cd toxicity may be at least partly attributed to the protection of the photosynthetic apparatus. It is well known that photoinhibition primarily results from overproduction of reactive oxygen species (ROS) through the photosynthetic electron transport chain under stress circumstances (Critchley, 1981). However, it needs to be further elucidated if Mg^2+^ protects the photosynthetic membrane from photo-oxidation by effectively scavenging ROS under lead stress conditions.

The influence of heavy metal on cellular organization is important for understanding physiological alterations under stress conditions (Souza et al., 2005). In the present study, chloroplasts were highly susceptible to stress induced by high lead conditions, as indicated by decreased lamellae, increased numbers of osmiophilic granules and disrupted thylakoid membranes (**Figure 5**). Damage to chloroplasts and thylakoid membranes in plants treated with heavy metals has been reported by Wu et al. (2014). We found that Mg^2+^ ameliorated the chloroplast ultrastructural disorders caused by lead (**Figure 5D**), which might explain the improved photosynthesis of the Mg-treated plants. Meanwhile, as also suggested by our study, the main processes underlying the improvement in plant photosynthesis induced by Mg^2+^ treatment are the enhanced light-use-efficiency and the protection of the chloroplast structures.

Many scientists have reported that Mg^2+^ supplementation enhances the tolerance to toxic metals by reducing the uptake and translocation of metals, including Cd^2+^ and Al^3+^ (Kashem and Kawai, 2007; Bose et al., 2011). We found that the accumulation of lead was greater in the roots than in the shoots of *T. grandis* seedlings (**Table 3**), indicating that the plants translocated lower concentrations of metals into the shoots (Yang et al., 1998). Higher Pb^2+^ concentrations in roots than shoots were observed in the Mg^2+^-alleviated plants, whereas the shoot Mg^2+^ concentrations were four-fold higher than the root Mg^2+^ concentrations in the Mg^2+^-alleviated plants (**Table 3**). These results suggest that Mg^2+^ application through the watering solution helped decrease the Pb^2+^ accumulation in the shoots. Similar findings have been reported by Kashem and Kawai (2007), who found that magnesium-alleviated plants showed decreased shoot Cd^2+^ concentration in Japanese mustard spinach (*Brassica rapa* L. var. *pervirdis*).

Furthermore, lead uptake significantly reduced total root length, surface area and volume compared with the control plants (**Table 1**). However, the application of Mg^2+^ increased the indices of root morphological traits of *T. grandis* seedlings under lead toxicity. Root oxidative activity implies the degree of root development and metabolic status (Liu et al., 2008). In the present study, lower root oxidative activity was found in *T. grandis* seedlings under Pb^2+^ toxicity than in the non-Pb^2+^-treated control plants, whereas higher root oxidative activity was observed in the Mg^2+^-alleviated plants than in the Pb^2+^-toxic plants (**Figure 4B**). These findings indicate that additional Mg^2+^ might increase the absorptive area of roots and, hence, increase the uptake of water and nutrients to improve plant growth (Glinka, 1980). This finding was consistent with the observed ultrastructure of the *T. grandis* seedlings roots. The Mg^2+^ application protected the integrity of the root cells, resulting in a visible nucleus, slightly condensed chromatin and irregularly swollen mitochondria with fractured and fuzzy cristae (**Figure 6**). Thus, Mg^2+^ application is an effective method to alleviate Pb^2+^ toxicity in *T. grandis* seedlings by improving root growth and root oxidative activity and protecting root ultrastructure.

## Conclusion

*Torreya grandis* seedlings exposed to 1400 mg kg^-1^ Pb^2+^ exhibited stress toxicity as indicated by reduced shoot growth. Mg^2+^ addition under Pb^2+^ stress conditions might have beneficial effects on the growth of *T. grandis* seedlings, as evidenced by increased shoot dry biomass, root dry biomass, chlorophyll contents, and photosynthesis as well as improved chloroplast ultrastructure. Moreover, additional Mg^2+^ in the solution containing Pb^2+^ decreased the Pb^2+^ concentration in the shoots and increased the Mg^2+^ concentration in the shoots. Furthermore, we showed that the positive effects of Mg^2+^ on the growth of *T. grandis* were triggered by protecting the morphology, activity and ultrastructure of the roots. To our knowledge, this study is the first study to show Mg^2+^-induced alleviation of lead toxicity in *T. grandis* seedlings and is of great importance for the cultivation of *T. grandis* seedlings in China, where soils are often contaminated with lead.

## Author Contributions

All authors listed, have made substantial, direct and intellectual contribution to the work, and approved it for publication.

## Conflict of Interest Statement

The authors declare that the research was conducted in the absence of any commercial or financial relationships that could be construed as a potential conflict of interest.

## Footnotes

1 www.regentinstruments.com
